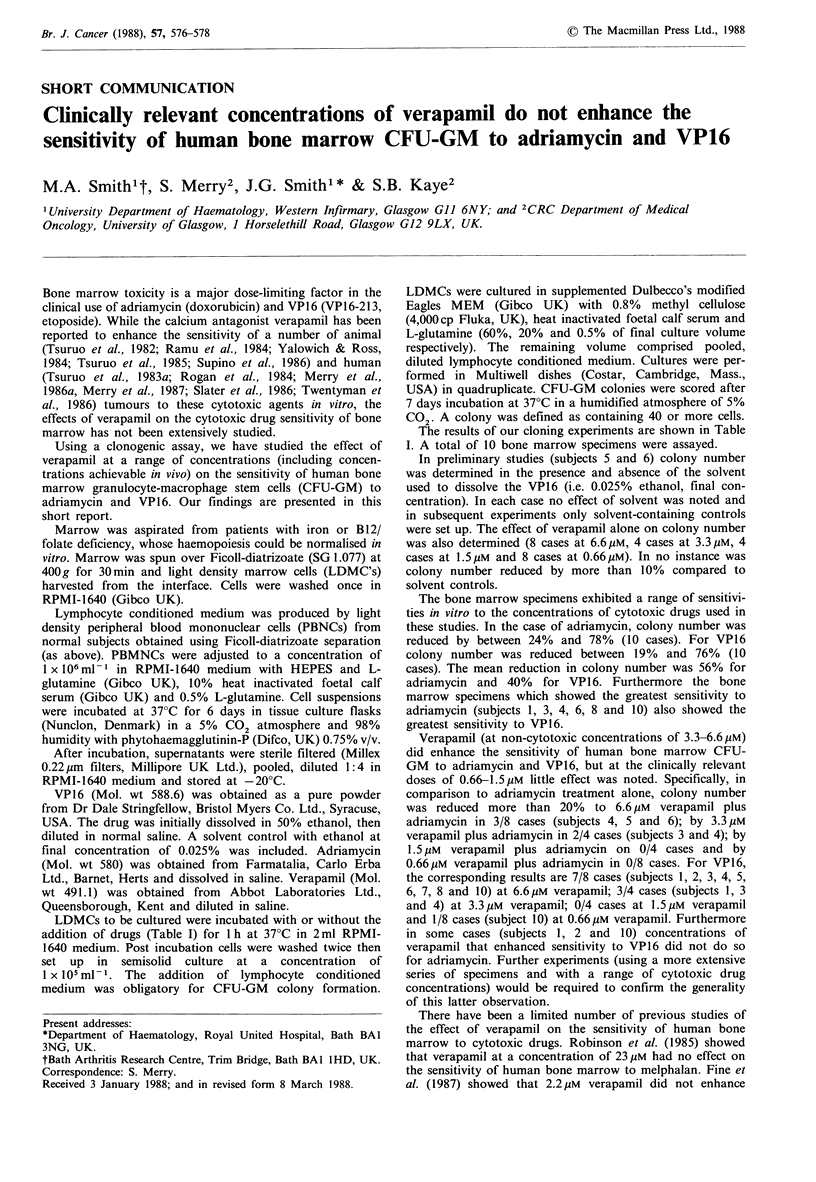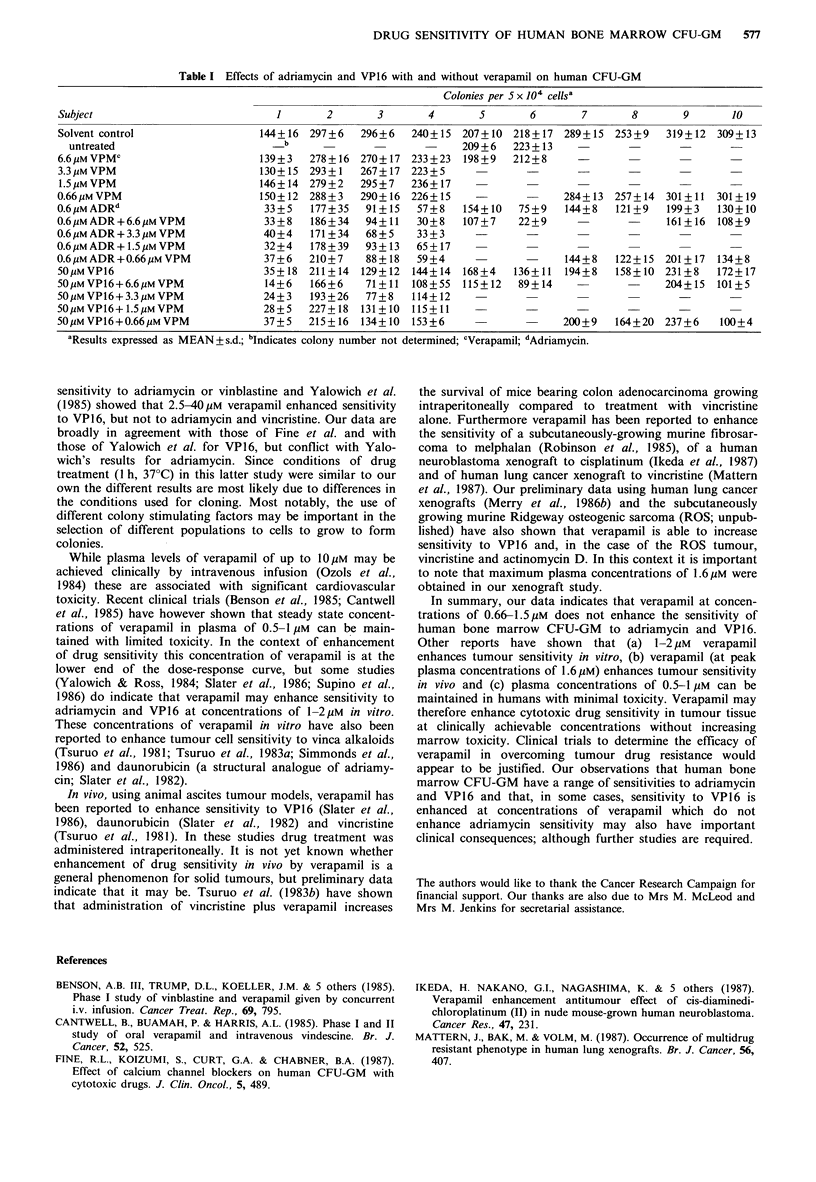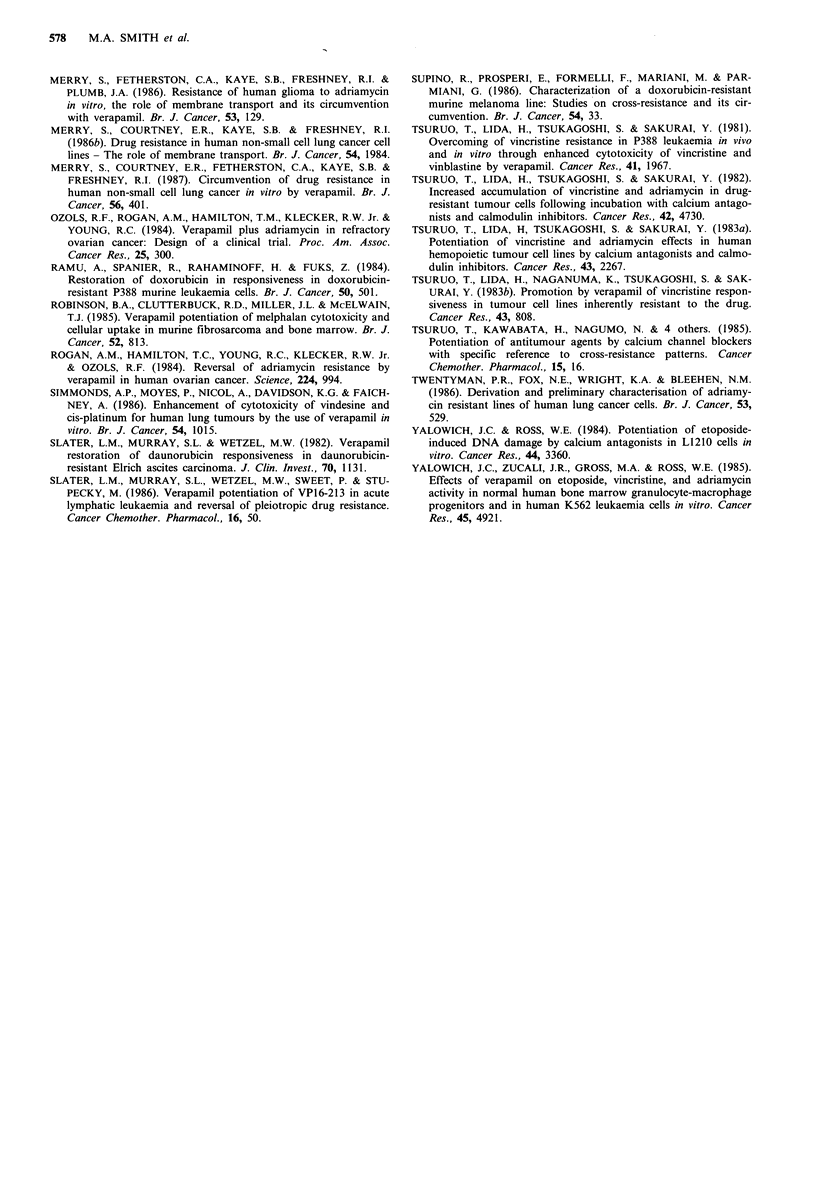# Clinically relevant concentrations of verapamil do not enhance the sensitivity of human bone marrow CFU-GM to adriamycin and VP16.

**DOI:** 10.1038/bjc.1988.131

**Published:** 1988-06

**Authors:** M. A. Smith, S. Merry, J. G. Smith, S. B. Kaye

**Affiliations:** University Department of Haematology, Western Infirmary, Glasgow, UK.


Br. J. Cancer (1988), 57, 576–578 © The Macmillan Press Ltd., 1988

SHORT COMMUNICATION

### Clinically relevant concentrations of verapamil do not enhance the sensitivity of human bone marrow CFU-GM to adriamycin and VP16

M.A. Smith[1]†, S. Merry[2], J.G. Smith[1]* & S.B. Kaye[2]

[1]*University Department of Haematology, Western Infirmary, Glasgow G11 6NY; and* [2]*CRC Department of Medical Oncology, University of Glasgow, 1 Horselethill Road, Glasgow G12 9LX, UK.*

Bone marrow toxicity is a major dose-limiting factor in the clinical use of adriamycin (doxorubicin) and VP16 (VP16-213, etoposide). While the calcium antagonist verapamil has been reported to enhance the sensitivity of a number of animal (Tsuruo *et al.*, 1982; Ramu *et al.*, 1984; Yalowich & Ross, 1984; Tsuruo *et al.*, 1985; Supino *et al.*, 1986) and human (Tsuruo *et al.*, 1983*a*; Rogan *et al.*, 1984; Merry *et al.*, 1986*a*, Merry *et al.*, 1987; Slater *et al.*, 1986; Twentyman *et al.*, 1986) tumours to these cytotoxic agents *in vitro*, the effects of verapamil on the cytotoxic drug sensitivity of bone marrow has not been extensively studied.

Using a clonogenic assay, we have studied the effect of verapamil at a range of concentrations (including concentrations achievable *in vivo*) on the sensitivity of human bone marrow granulocyte-macrophage stem cells (CFU-GM) to adriamycin and VP16. Our findings are presented in this short report.

Marrow was aspirated from patients with iron or B12/folate deficiency, whose haemopoiesis could be normalised *in vitro*. Marrow was spun over Ficoll-diatrizoate (SG 1.077) at 400*g* for 30 min and light density marrow cells (LDMC's) harvested from the interface. Cells were washed once in RPMI-1640 (Gibco UK).

Lymphocyte conditioned medium was produced by light density peripheral blood mononuclear cells (PBNCs) from normal subjects obtained using Ficoll-diatrizoate separation (as above). PBMNCs were adjusted to a concentration of $1\times10^6\,ml^{-1}$ in RPMI-1640 medium with HEPES and L-glutamine (Gibco UK), 10% heat inactivated foetal calf serum (Gibco UK) and 0.5% L-glutamine. Cell suspensions were incubated at 37°C for 6 days in tissue culture flasks (Nunclon, Denmark) in a 5% $CO_2$ atmosphere and 98% humidity with phytohaemagglutinin-P (Difco, UK) 0.75% v/v. After incubation, supernatants were sterile filtered (Millex 0.22 μm filters, Millipore UK Ltd.), pooled, diluted 1:4 in RPMI-1640 medium and stored at −20°C.

VP16 (Mol. wt 588.6) was obtained as a pure powder from Dr Dale Stringfellow, Bristol Myers Co. Ltd., Syracuse, USA. The drug was initially dissolved in 50% ethanol, then diluted in normal saline. A solvent control with ethanol at final concentration of 0.025% was included. Adriamycin (Mol. wt 580) was obtained from Farmatalia, Carlo Erba Ltd., Barnet, Herts and dissolved in saline. Verapamil (Mol. wt 491.1) was obtained from Abbot Laboratories Ltd., Queensborough, Kent and diluted in saline.

LDMCs to be cultured were incubated with or without the addition of drugs (Table I) for 1 h at 37°C in 2 ml RPMI-1640 medium. Post incubation cells were washed twice then set up in semisolid culture at a concentration of $1\times10^5\,ml^{-1}$. The addition of lymphocyte conditioned medium was obligatory for CFU-GM colony formation. LDMCs were cultured in supplemented Dulbecco's modified Eagles MEM (Gibco UK) with 0.8% methyl cellulose (4,000 cp Fluka, UK), heat inactivated foetal calf serum and L-glutamine (60%, 20% and 0.5% of final culture volume respectively). The remaining volume comprised pooled, diluted lymphocyte conditioned medium. Cultures were performed in Multiwell dishes (Costar, Cambridge, Mass., USA) in quadruplicate. CFU-GM colonies were scored after 7 days incubation at 37°C in a humidified atmosphere of 5% $CO_2$. A colony was defined as containing 40 or more cells.

The results of our cloning experiments are shown in Table I. A total of 10 bone marrow specimens were assayed.

In preliminary studies (subjects 5 and 6) colony number was determined in the presence and absence of the solvent used to dissolve the VP16 (i.e. 0.025% ethanol, final concentration). In each case no effect of solvent was noted and in subsequent experiments only solvent-containing controls were set up. The effect of verapamil alone on colony number was also determined (8 cases at 6.6 μM, 4 cases at 3.3 μM, 4 cases at 1.5 μM and 8 cases at 0.66 μM). In no instance was colony number reduced by more than 10% compared to solvent controls.

The bone marrow specimens exhibited a range of sensitivities *in vitro* to the concentrations of cytotoxic drugs used in these studies. In the case of adriamycin, colony number was reduced by between 24% and 78% (10 cases). For VP16 colony number was reduced between 19% and 76% (10 cases). The mean reduction in colony number was 56% for adriamycin and 40% for VP16. Furthermore the bone marrow specimens which showed the greatest sensitivity to adriamycin (subjects 1, 3, 4, 6, 8 and 10) also showed the greatest sensitivity to VP16.

Verapamil (at non-cytotoxic concentrations of 3.3–6.6 μM) did enhance the sensitivity of human bone marrow CFU-GM to adriamycin and VP16, but at the clinically relevant doses of 0.66–1.5 μM little effect was noted. Specifically, in comparison to adriamycin treatment alone, colony number was reduced more than 20% to 6.6 μM verapamil plus adriamycin in 3/8 cases (subjects 4, 5 and 6); by 3.3 μM verapamil plus adriamycin in 2/4 cases (subjects 3 and 4); by 1.5 μM verapamil plus adriamycin on 0/4 cases and by 0.66 μM verapamil plus adriamycin in 0/8 cases. For VP16, the corresponding results are 7/8 cases (subjects 1, 2, 3, 4, 5, 6, 7, 8 and 10) at 6.6 μM verapamil; 3/4 cases (subjects 1, 3 and 4) at 3.3 μM verapamil; 0/4 cases at 1.5 μM verapamil and 1/8 cases (subject 10) at 0.66 μM verapamil. Furthermore in some cases (subjects 1, 2 and 10) concentrations of verapamil that enhanced sensitivity to VP16 did not do so for adriamycin. Further experiments (using a more extensive series of specimens and with a range of cytotoxic drug concentrations) would be required to confirm the generality of this latter observation.

There have been a limited number of previous studies of the effect of verapamil on the sensitivity of human bone marrow to cytotoxic drugs. Robinson *et al.* (1985) showed that verapamil at a concentration of 23 μM had no effect on the sensitivity of human bone marrow to melphalan. Fine *et al.* (1987) showed that 2.2 μM verapamil did not enhance

Present addresses:
*Department of Haematology, Royal United Hospital, Bath BA1 3NG, UK.
†Bath Arthritis Research Centre, Trim Bridge, Bath BA1 1HD, UK.
Correspondence: S. Merry.
Received 3 January 1988; and in revised form 8 March 1988.

**Table I** Effects of adriamycin and VP16 with and without verapamil on human CFU-GM

| | *Colonies per $5 \times 10^4$ cells*[a] | | | | | | | | | |
|---|---|---|---|---|---|---|---|---|---|---|
| *Subject* | *1* | *2* | *3* | *4* | *5* | *6* | *7* | *8* | *9* | *10* |
| Solvent control | 144±16 | 297±6 | 296±6 | 240±15 | 207±10 | 218±17 | 289±15 | 253±9 | 319±12 | 309±13 |
| untreated | —[b] | — | — | — | 209±6 | 223±13 | — | — | — | — |
| 6.6 μM VPM[c] | 139±3 | 278±16 | 270±17 | 233±23 | 198±9 | 212±8 | — | — | — | — |
| 3.3 μM VPM | 130±15 | 293±1 | 267±17 | 223±5 | — | — | — | — | — | — |
| 1.5 μM VPM | 146±14 | 279±2 | 295±7 | 236±17 | — | — | — | — | — | — |
| 0.66 μM VPM | 150±12 | 288±3 | 290±16 | 226±15 | — | — | 284±13 | 257±14 | 301±11 | 301±19 |
| 0.6 μM ADR[d] | 33±5 | 177±35 | 91±15 | 57±8 | 154±10 | 75±9 | 144±8 | 121±9 | 199±3 | 130±10 |
| 0.6 μM ADR + 6.6 μM VPM | 33±8 | 186±34 | 94±11 | 30±8 | 107±7 | 22±9 | — | — | 161±16 | 108±9 |
| 0.6 μM ADR + 3.3 μM VPM | 40±4 | 171±34 | 68±5 | 33±3 | — | — | — | — | — | — |
| 0.6 μM ADR + 1.5 μM VPM | 32±4 | 178±39 | 93±13 | 65±17 | — | — | — | — | — | — |
| 0.6 μM ADR + 0.66 μM VPM | 37±6 | 210±7 | 88±18 | 59±4 | — | — | 144±8 | 122±15 | 201±17 | 134±8 |
| 50 μM VP16 | 35±18 | 211±14 | 129±12 | 144±14 | 168±4 | 136±11 | 194±8 | 158±10 | 231±8 | 172±17 |
| 50 μM VP16 + 6.6 μM VPM | 14±6 | 166±6 | 71±11 | 108±55 | 115±12 | 89±14 | — | — | 204±15 | 101±5 |
| 50 μM VP16 + 3.3 μM VPM | 24±3 | 193±26 | 77±8 | 114±12 | — | — | — | — | — | — |
| 50 μM VP16 + 1.5 μM VPM | 28±5 | 227±18 | 131±10 | 115±11 | — | — | — | — | — | — |
| 50 μM VP16 + 0.66 μM VPM | 37±5 | 215±16 | 134±10 | 153±6 | — | — | 200±9 | 164±20 | 237±6 | 100±4 |

[a]Results expressed as MEAN±s.d.; [b]Indicates colony number not determined; [c]Verapamil; [d]Adriamycin.

sensitivity to adriamycin or vinblastine and Yalowich *et al.* (1985) showed that 2.5–40 μM verapamil enhanced sensitivity to VP16, but not to adriamycin and vincristine. Our data are broadly in agreement with those of Fine *et al.* and with those of Yalowich *et al.* for VP16, but conflict with Yalowich's results for adriamycin. Since conditions of drug treatment (1 h, 37°C) in this latter study were similar to our own the different results are most likely due to differences in the conditions used for cloning. Most notably, the use of different colony stimulating factors may be important in the selection of different populations to cells to grow to form colonies.

While plasma levels of verapamil of up to 10 μM may be achieved clinically by intravenous infusion (Ozols *et al.*, 1984) these are associated with significant cardiovascular toxicity. Recent clinical trials (Benson *et al.*, 1985; Cantwell *et al.*, 1985) have however shown that steady state concentrations of verapamil in plasma of 0.5–1 μM can be maintained with limited toxicity. In the context of enhancement of drug sensitivity this concentration of verapamil is at the lower end of the dose-response curve, but some studies (Yalowich & Ross, 1984; Slater *et al.*, 1986; Supino *et al.*, 1986) do indicate that verapamil may enhance sensitivity to adriamycin and VP16 at concentrations of 1–2 μM *in vitro*. These concentrations of verapamil *in vitro* have also been reported to enhance tumour cell sensitivity to vinca alkaloids (Tsuruo *et al.*, 1981; Tsuruo *et al.*, 1983*a*; Simmonds *et al.*, 1986) and daunorubicin (a structural analogue of adriamycin; Slater *et al.*, 1982).

*In vivo*, using animal ascites tumour models, verapamil has been reported to enhance sensitivity to VP16 (Slater *et al.*, 1986), daunorubicin (Slater *et al.*, 1982) and vincristine (Tsuruo *et al.*, 1981). In these studies drug treatment was administered intraperitoneally. It is not yet known whether enhancement of drug sensitivity *in vivo* by verapamil is a general phenomenon for solid tumours, but preliminary data indicate that it may be. Tsuruo *et al.* (1983*b*) have shown that administration of vincristine plus verapamil increases the survival of mice bearing colon adenocarcinoma growing intraperitoneally compared to treatment with vincristine alone. Furthermore verapamil has been reported to enhance the sensitivity of a subcutaneously-growing murine fibrosarcoma to melphalan (Robinson *et al.*, 1985), of a human neuroblastoma xenograft to cisplatinum (Ikeda *et al.*, 1987) and of human lung cancer xenograft to vincristine (Mattern *et al.*, 1987). Our preliminary data using human lung cancer xenografts (Merry *et al.*, 1986*b*) and the subcutaneously growing murine Ridgeway osteogenic sarcoma (ROS; unpublished) have also shown that verapamil is able to increase sensitivity to VP16 and, in the case of the ROS tumour, vincristine and actinomycin D. In this context it is important to note that maximum plasma concentrations of 1.6 μM were obtained in our xenograft study.

In summary, our data indicates that verapamil at concentrations of 0.66–1.5 μM does not enhance the sensitivity of human bone marrow CFU-GM to adriamycin and VP16. Other reports have shown that (a) 1–2 μM verapamil enhances tumour sensitivity *in vitro*, (b) verapamil (at peak plasma concentrations of 1.6 μM) enhances tumour sensitivity *in vivo* and (c) plasma concentrations of 0.5–1 μM can be maintained in humans with minimal toxicity. Verapamil may therefore enhance cytotoxic drug sensitivity in tumour tissue at clinically achievable concentrations without increasing marrow toxicity. Clinical trials to determine the efficacy of verapamil in overcoming tumour drug resistance would appear to be justified. Our observations that human bone marrow CFU-GM have a range of sensitivities to adriamycin and VP16 and that, in some cases, sensitivity to VP16 is enhanced at concentrations of verapamil which do not enhance adriamycin sensitivity may also have important clinical consequences; although further studies are required.

The authors would like to thank the Cancer Research Campaign for financial support. Our thanks are also due to Mrs M. McLeod and Mrs M. Jenkins for secretarial assistance.

### References

BENSON, A.B. III, TRUMP, D.L., KOELLER, J.M. & 5 others (1985). Phase I study of vinblastine and verapamil given by concurrent i.v. infusion. *Cancer Treat. Rep.*, **69**, 795.

CANTWELL, B., BUAMAH, P. & HARRIS, A.L. (1985). Phase I and II study of oral verapamil and intravenous vindescine. *Br. J. Cancer*, **52**, 525.

FINE, R.L., KOIZUMI, S., CURT, G.A. & CHABNER, B.A. (1987). Effect of calcium channel blockers on human CFU-GM with cytotoxic drugs. *J. Clin. Oncol.*, **5**, 489.

IKEDA, H. NAKANO, G.I., NAGASHIMA, K. & 5 others (1987). Verapamil enhancement antitumour effect of cis-diaminedichloroplatinum (II) in nude mouse-grown human neuroblastoma. *Cancer Res.*, **47**, 231.

MATTERN, J., BAK, M. & VOLM, M. (1987). Occurrence of multidrug resistant phenotype in human lung xenografts. *Br. J. Cancer*, **56**, 407.

MERRY, S., FETHERSTON, C.A., KAYE, S.B., FRESHNEY, R.I. & PLUMB, J.A. (1986). Resistance of human glioma to adriamycin *in vitro*, the role of membrane transport and its circumvention with verapamil. *Br. J. Cancer*, **53**, 129.

MERRY, S., COURTNEY, E.R., KAYE, S.B. & FRESHNEY, R.I. (1986*b*). Drug resistance in human non-small cell lung cancer cell lines – The role of membrane transport. *Br. J. Cancer*, **54**, 1984.

MERRY, S., COURTNEY, E.R., FETHERSTON, C.A., KAYE, S.B. & FRESHNEY, R.I. (1987). Circumvention of drug resistance in human non-small cell lung cancer *in vitro* by verapamil. *Br. J. Cancer*, **56**, 401.

OZOLS, R.F., ROGAN, A.M., HAMILTON, T.M., KLECKER, R.W. Jr. & YOUNG, R.C. (1984). Verapamil plus adriamycin in refractory ovarian cancer: Design of a clinical trial. *Proc. Am. Assoc. Cancer Res.*, **25**, 300.

RAMU, A., SPANIER, R., RAHAMINOFF, H. & FUKS, Z. (1984). Restoration of doxorubicin in responsiveness in doxorubicin-resistant P388 murine leukaemia cells. *Br. J. Cancer*, **50**, 501.

ROBINSON, B.A., CLUTTERBUCK, R.D., MILLER, J.L. & McELWAIN, T.J. (1985). Verapamil potentiation of melphalan cytotoxicity and cellular uptake in murine fibrosarcoma and bone marrow. *Br. J. Cancer*, **52**, 813.

ROGAN, A.M., HAMILTON, T.C., YOUNG, R.C., KLECKER, R.W. Jr. & OZOLS, R.F. (1984). Reversal of adriamycin resistance by verapamil in human ovarian cancer. *Science*, **224**, 994.

SIMMONDS, A.P., MOYES, P., NICOL, A., DAVIDSON, K.G. & FAICHNEY, A. (1986). Enhancement of cytotoxicity of vindesine and cis-platinum for human lung tumours by the use of verapamil *in vitro*. *Br. J. Cancer*, **54**, 1015.

SLATER, L.M., MURRAY, S.L. & WETZEL, M.W. (1982). Verapamil restoration of daunorubicin responsiveness in daunorubicin-resistant Elrich ascites carcinoma. *J. Clin. Invest.*, **70**, 1131.

SLATER, L.M., MURRAY, S.L., WETZEL, M.W., SWEET, P. & STUPECKY, M. (1986). Verapamil potentiation of VP16-213 in acute lymphatic leukaemia and reversal of pleiotropic drug resistance. *Cancer Chemother. Pharmacol.*, **16**, 50.

SUPINO, R., PROSPERI, E., FORMELLI, F., MARIANI, M. & PARMIANI, G. (1986). Characterization of a doxorubicin-resistant murine melanoma line: Studies on cross-resistance and its circumvention. *Br. J. Cancer*, **54**, 33.

TSURUO, T., LIDA, H., TSUKAGOSHI, S. & SAKURAI, Y. (1981). Overcoming of vincristine resistance in P388 leukaemia *in vivo* and *in vitro* through enhanced cytotoxicity of vincristine and vinblastine by verapamil. *Cancer Res.*, **41**, 1967.

TSURUO, T., LIDA, H., TSUKAGOSHI, S. & SAKURAI, Y. (1982). Increased accumulation of vincristine and adriamycin in drug-resistant tumour cells following incubation with calcium antagonists and calmodulin inhibitors. *Cancer Res.*, **42**, 4730.

TSURUO, T., LIDA, H, TSUKAGOSHI, S. & SAKURAI, Y. (1983*a*). Potentiation of vincristine and adriamycin effects in human hemopoietic tumour cell lines by calcium antagonists and calmodulin inhibitors. *Cancer Res.*, **43**, 2267.

TSURUO, T., LIDA, H., NAGANUMA, K., TSUKAGOSHI, S. & SAKURAI, Y. (1983*b*). Promotion by verapamil of vincristine responsiveness in tumour cell lines inherently resistant to the drug. *Cancer Res.*, **43**, 808.

TSURUO, T., KAWABATA, H., NAGUMO, N. & 4 others. (1985). Potentiation of antitumour agents by calcium channel blockers with specific reference to cross-resistance patterns. *Cancer Chemother. Pharmacol.*, **15**, 16.

TWENTYMAN, P.R., FOX, N.E., WRIGHT, K.A. & BLEEHEN, N.M. (1986). Derivation and preliminary characterisation of adriamycin resistant lines of human lung cancer cells. *Br. J. Cancer*, **53**, 529.

YALOWICH, J.C. & ROSS, W.E. (1984). Potentiation of etoposide-induced DNA damage by calcium antagonists in L1210 cells *in vitro*. *Cancer Res.*, **44**, 3360.

YALOWICH, J.C., ZUCALI, J.R., GROSS, M.A. & ROSS, W.E. (1985). Effects of verapamil on etoposide, vincristine, and adriamycin activity in normal human bone marrow granulocyte-macrophage progenitors and in human K562 leukaemia cells *in vitro*. *Cancer Res.*, **45**, 4921.